# Mandibular Osteoblastoma: A Case Report of a Rare Entity

**DOI:** 10.7759/cureus.53900

**Published:** 2024-02-09

**Authors:** George Skariah P, Sarath S S, Kavya P Valsaraj, Sherin Shahana, Syed S Basha, Abhishek Pathak

**Affiliations:** 1 Oral and Maxillofacial Surgery, PMS Hospital of Dental Sciences and Research, Vattappara, Trivandrum, IND; 2 Oral and Maxillofacial Surgery, Government Dental College, Trivandrum, IND

**Keywords:** bone‑forming tumor, osteoid tumor, orthopantomogram, maxillofacial skeleton, benign osteoblastoma

## Abstract

Osteoblastoma, an uncommon bone neoplasm characterized by the formation of osteoid tissue, constitutes a rare subset of bone tumors, representing only a negligible percentage of cases. While extensive research on the condition has identified a predilection for long bones and vertebrae, occurrences in facial bones are infrequent. This case report discusses a unique presentation in a nine-year-old female diagnosed with a tumor located in the mandibular parasymphysis region. Radiological examination revealed distinctive irregular contours surrounding the lesion, prompting a comprehensive investigation involving biopsy and subsequent histological analysis. The ensuing evaluation definitively confirmed the diagnosis of a typical benign osteoblastoma. This report highlights the novelty of an unresected benign osteoblastoma situated in the mandible, currently undergoing periodic review, with the option of surgery also on the table.

## Introduction

Benign osteoblastoma, a tumor characterized by the formation of osteoid and bone, rarely involves facial bones. It was first reported by Jaffe and Mayer in 1932 [1], with the clinical term being formally established in 1956 [2,3]. Despite its rarity, this tumor accounts for approximately 1% of primary bone tumors affecting the entire body [4]. Notably, benign osteoblastoma tends to manifest in the maxillofacial skeleton, with around 15% of cases presenting in this region and a higher incidence in the mandible [5,6]. Borello and Sedano played a pivotal role in framing the early understanding of this condition, reporting the first case of this lesion in 1967 [7]. Despite its recognition, cases of osteoblastoma diagnosed specifically in the jaws are infrequently documented.

In this report, we present a rare case of osteoblastoma in a nine-year-old female. The tumor was situated in the posterior region of her lower jaw, further emphasizing the unique nature of this pathology, particularly in pediatric patients. The intricate details of this case presented here contribute to the growing body of knowledge on benign osteoblastoma and its diverse clinical presentations.

## Case presentation

A nine-year-old female presented to the Department of Oral and Maxillofacial Surgery with a two-week history of pain and swelling on the left side of her face. Given that similar symptoms had occurred one year prior, which had responded favorably to oral antibiotics and analgesics, a decision was made to pursue a course of antibiotics initially. This approach provided mild relief for one week. Of note, the clinical presentation in our case varied from the typical manifestations of osteoblastoma, which generally involve indolent, dull, aching pain resistant to nonsteroidal anti-inflammatory drugs (NSAIDs). Despite this deviation from the usual pattern, the patient's symptoms significantly improved with the prescribed oral antibiotics and analgesics. This strategy aimed to address the immediate symptoms and relieve the patient while further investigations, including radiological and histopathological assessments, were planned to reach a definitive diagnosis. The decision to use antibiotics was based on the successful resolution of similar symptoms in the patient's previous episode. It was intended as a pragmatic approach to managing the acute phase of the condition pending further diagnostic evaluation.

Upon examination, an extraoral swelling approximately 6 x 8 cm in size and affecting the left body of the mandible was observed. The swelling extended from the parasymphyseal region anteriorly to the right mandibular second premolar posteriorly, with smooth overlying skin. This swelling induced noticeable facial asymmetry, accompanied by palpable and tender cervical lymph nodes. Intraorally, no significant abnormalities were noted, and the overlying mucosa appeared normal. Laboratory tests, including blood routine values, liver function, and renal function, were within normal limits, except for elevated erythrocyte sedimentation rate (ESR) levels.

**Figure 1:**
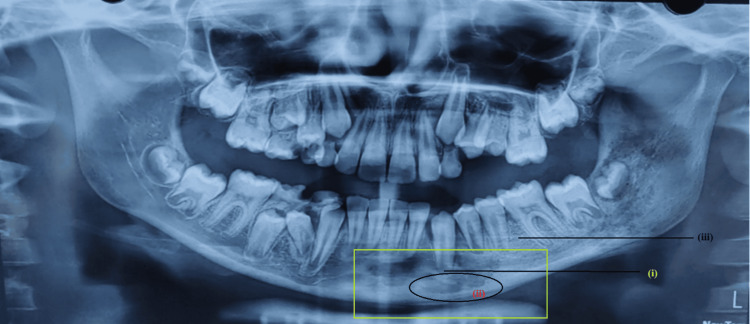
Orthopantomogram showing a mixed radiolucent radiopaque lesion in the left parasymphyseal region extending from #41 to #35 (i) Erosion of the lower cortical border of the mandible noted in relation to erupting #33. (ii) The evident loss of cortication of the follicle around the developing roots of #33, #34, and #35. (iii) Altered trabecular pattern observed in relation to the periapical region of #35 and #36 #: tooth number

An orthopantomogram (Figure 1) revealed a mixed radiolucent-radiopaque lesion in the left parasymphyseal region, crossing the midline from the medial aspect of the root of #41 on the right side to the medial aspect of the root of #35 on the left side. The lesion exhibited irregular margins, and erosion of the lower cortical border of the mandible was evident about erupting #33. Additionally, there was a loss of cortication of the follicle around the developing roots of #33, #34, and #35. Posterior to the lesion, an altered trabecular pattern was observed about the periapical region of #35 and #36. Widening of the periodontal ligament space was noted with #36. A diffuse lytic lesion with irregular margins extending posteriorly, connecting the angle and mandibular ramus to the sigmoid notch, was observed posterior to #36. The loss of cortication of the superior and inferior borders of the mandibular canal was noted, along with the loss of cortication of the follicle with #37 and #38. Radiographic features suggested a chronic destructive lesion resembling osteomyelitis rather than osteoma or osteoblastoma. Tooth vitality testing (sensitivity to cold) revealed no insensitivity in the teeth within the affected region.

After the lesion underwent an incision biopsy under local anesthesia, the patient experienced a subsequent episode of pain and swelling 15 days post-biopsy. This episode was effectively managed through the administration of oral antibiotics and analgesics. Thorough assessments, including liver function tests, renal function tests, C-reactive protein (CRP) levels, and parathyroid values, returned results within normal limits. The incisional biopsy report indicated an inflammatory lesion with foci of bone necrosis; however, a definitive diagnosis remained elusive.

**Figure 2:**
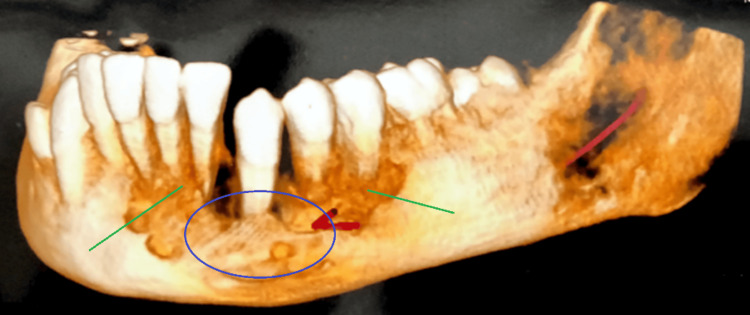
CT scan showing permeative pattern of bone destruction involving the left ramus of the mandible and from #41 to #35 The areas marked show specific regions of bone destruction CT: computed tomography

A CT scan (Figure 2) unveiled features consistent with chronic sclerosing osteomyelitis. This was manifested by subperiosteal bone formation in angles #35 to #38 and the ramus region. The multi-slice CT scan further elucidated the lytic nature of the lesion, marked by a permeative pattern of bone destruction involving the mandibular ramus on the left side. Additionally, periosteal thickening and hypodensity of the left masseter and left temporalis were also observed. The differential diagnoses spanned a spectrum of conditions, encompassing chronic sclerosing osteomyelitis, osteosarcoma, metastatic deposits, fibro-osseous lesions like fibrous dysplasia, ossifying fibroma, cementoblastoma, and osteoid osteoma, among others. The intricacy of these findings underscores the challenges inherent in reaching a definitive diagnosis in these cases and accentuates the need for further evaluation and contemplation of various potential pathologies.

**Figure 3:**
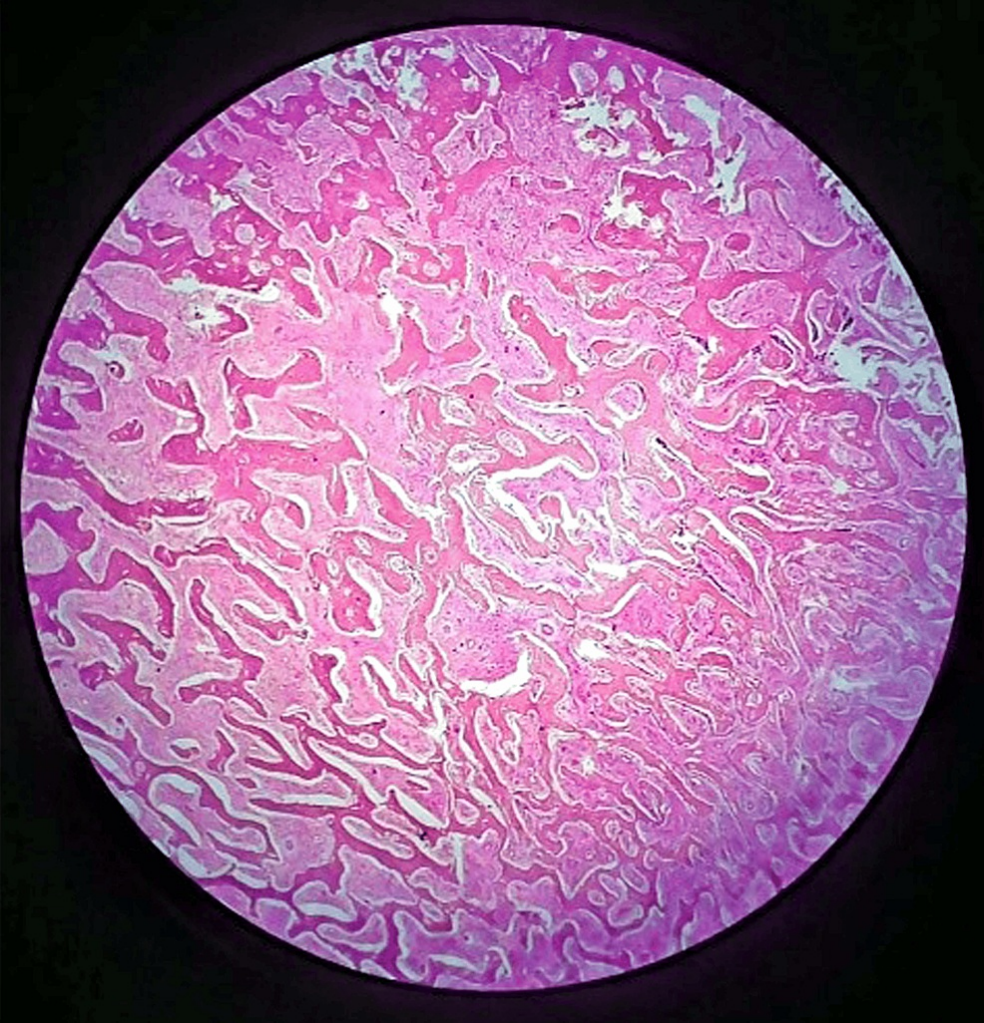
Photomicrograph of osteoblastoma showing haphazard proliferation of interlacing trabeculae in a fibrovascular stroma

**Figure 4:**
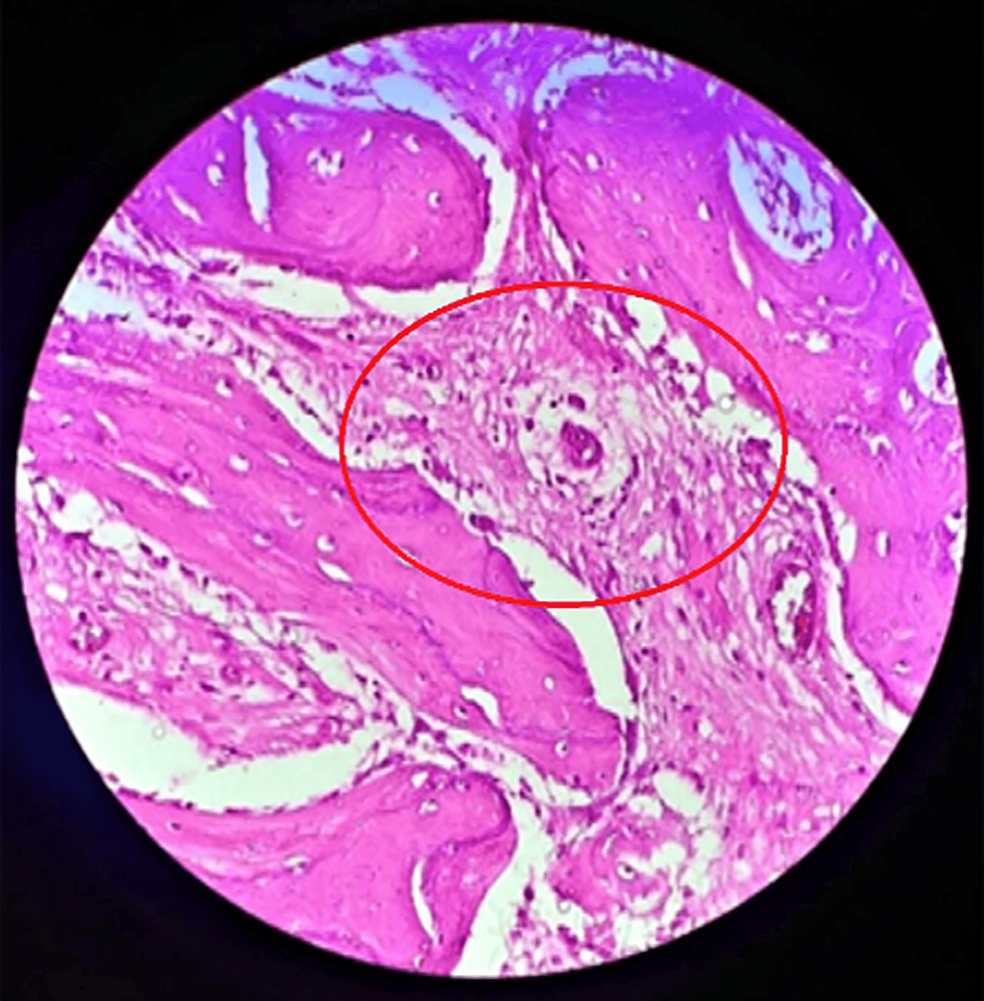
Photomicrograph of osteoblastoma rimmed by a layer of osteoblasts and tiny foci of intralesional hemorrhage The circled area shows foci of intralesional hemorrhage

Two weeks later, a second incision biopsy was performed under general anesthesia. Histopathological examination revealed a haphazard proliferation of interlacing trabeculae, as depicted in Figure 3, along with osteoblastic rimming by benign activated osteoblasts, as evidenced in Figure 4. The microscopic analysis also identified abnormal mitotic figures and dysplastic features. Collectively, these histopathologic features were consistent with the diagnosis of osteoblastoma. This conclusive finding clarifies the lesion's nature, marking a significant milestone in accurately characterizing and understanding the underlying pathology.

## Discussion

Osteoblastoma primarily targets the posterior regions of the mandible, displaying a distinct male predilection with a male-to-female ratio of 2:1. This lesion is notably prevalent in individuals under the age of 20 years, constituting approximately 75% of cases, with an average age of onset at 18 years [8]. The typical size of the lesion falls within the range of 2-4 cm but can extend up to 10 cm [9]. The clinical prognosis of osteoblastoma varies based on factors such as recurrence, behavioral characteristics, and the potential for sarcomatous changes [10]. The radiographic examination of our patient revealed an absence of a well-defined border, suggesting that a clear plane of cleavage separating the lesion from normal bone might not be identified during surgery. Surgical intervention in such cases would have necessitated the extraction of six lower anterior teeth, lower left posterior teeth, and resection. Conventional osteoblastoma typically does not warrant radiation therapy or chemotherapy, even if the tumor shows apparent responsiveness to chemotherapy [11]. However, in situations where surgical excision is contraindicated or in the likelihood of aggression or recurrence, radiation therapy may be considered [12].

Surgical excision remains the preferred method for managing osteoblastoma, with alternative approaches including curettage or local excision [13]. Recurrence following surgery is rare, and block marginal resection provides an advantage in limiting recurrences [14,15]. En-bloc resection is seldom performed nowadays. The prognosis of osteoblastoma is contingent on the recurrence rate, which can occur in up to 50% of cases [16,17]. Following tumor curettage, bone grafts can be employed for bone regeneration [4]. In our case, given the patient's age and the potential for surgical deformity, consultations with multiple specialties were conducted. A collective decision was made to defer surgery for the time being, opting instead for periodic follow-up every month. The option for surgery remains open, pending substantial radiographic or clinical reasons that may necessitate intervention with a surgeon's scalpel. This approach highlights the importance of ongoing evaluation and emphasizes a cautious and patient-centric strategy in managing this condition.

## Conclusions

The rarity of osteoblastoma necessitates a meticulous approach to differentiate it from other bone neoplasms to establish a definitive diagnosis. A comprehensive understanding and correlation of its distinct features are crucial for accurate identification and effective management. Diagnostic tools such as orthopantomograms, CT scans, and histopathological assessments are critical in making early detection and establishing a prognosis, as demonstrated in the current case. These diagnostic modalities not only aid in demonstrating the unique characteristics of the lesion but also guide clinicians in making informed decisions regarding suitable treatment strategies. The importance of a thorough diagnostic process cannot be overstated in ensuring optimal outcomes for patients presenting with osteoblastoma.
